# Differences in Food Craving in Individuals With Obesity With and Without Binge Eating Disorder

**DOI:** 10.3389/fpsyg.2021.660880

**Published:** 2021-06-02

**Authors:** Janina Reents, Anya Pedersen

**Affiliations:** Institut für Psychologie, Philosophische Fakultät, Christian-Albrechts-Universität zu Kiel, Kiel, Germany

**Keywords:** overeating, cue reactivity, obesogenic environment, food cue exposure, HFHS diet, cue exposure therapy, emotional eating

## Abstract

Overeating behavior is supposedly a major contributing factor to weight gain and obesity. Binge eating disorder (BED) with reoccurring episodes of excessive overeating is strongly associated with obesity. Learning models of overeating behavior and BED assume that mere confrontation with food leads to a conditioned response that is experienced as food craving. Accordingly, individuals with obesity and BED were shown to have high trait food cravings. To date, little is known about differences in state food cravings and cue reactivity at the sight of palatable food in individuals with obesity and BED compared to individuals with obesity without BED. Therefore, the aim of our study was to examine differences in cue-induced, state and trait food cravings in people with obesity with and without BED. We found that all aspects of food cravings were more prevalent in individuals with obesity and BED than in individuals without BED. By implementing a food cue reactivity paradigm, our results show that individuals with obesity with BED have more cue-induced cravings than individuals with obesity without BED. Moreover, these cue-induced cravings in individuals with obesity and BED were highest for high-fat and high-sugar foods as opposed to low-calorie foods. Thus, our results emphasize the role of increased cue reactivity and craving at the sight of palatable foods in individuals with obesity and BED. Hence, our findings support etiological models of conditioned binge eating and are in line with interventions targeting cue reactivity in BED.

## Introduction

Overeating, i.e., consumption of food in the absence of physiological need with an inability to reduce ingestion, is one relevant factor in the complex etiology of obesity (Moore et al., 2017; McCuen-Wurst et al., 2018). If overeating is characterized by the consumption of an unusually large amount of food in a short duration and/or the individual experiences a loss of control over ingestion, this is considered as binge eating (for a review see Meany et al., 2014). In treatment programs for obesity about 5–30% of patients report such behavior and fulfill further criteria for the diagnosis of binge eating disorder (BED; DSM-5, de Zwaan, 2001; Abilés et al., 2010; American Psychiatric Association, 2013), which negatively affects obesity treatment outcomes (Meany et al., 2014; Chao et al., 2016). According to DSM-5 (American Psychiatric Association, 2013), episodes of binge eating must reoccur at least once per week for 3 months and are not accompanied by regular compensatory behavior. At least three of the following characteristics appear in such binge eating episodes: eating until uncomfortably full, eating large amounts in the absence of hunger, consuming food alone to avoid embarrassment, feeling disgust, sadness, or guilt because of the behavior. Moreover, a significant amount of distress must be associated with the binge episodes. Hence, criteria acknowledge an association between binge eating and negative emotional states. Accordingly, the comorbidity rate of BED and depressive disorders is high (Man Lapidoth et al., 2011) and associated with poorer weight loss outcomes in clinical treatments (Pagoto et al., 2007).

Overeating behavior in general, and binge eating in particular have both been linked to food craving (Sobik et al., 2005; White and Grilo, 2005; Chao et al., 2014; Innamorati et al., 2014; Oliveira and Cordás, 2020), i.e., the intense desire to eat specific food (Weingarten and Elston, 1990; Hill, 2007). Food cravings are accountable for up to 11% of the variance in weight gain and they are positively associated with BMI (for a meta-analysis see Boswell and Kober, 2016). Concerning the etiology of binge eating, Jansen (1998) suggests a cue-induced food craving leading to excessive ingestion. This conditional model of binge eating proposes environmental or interoceptive stimuli (e.g., the sight of food, emotions) to trigger a conditioned autonomic response (e.g., increases in salivary flow and heart rate), which is experienced as food craving. Meyer et al. (2015) showed the first evidence of differential acquisition of conditioned responses to food cues in overweight and lean individuals. The authors paired non-eating related visual cues with chocolate milk and with tasteless water, respectively. They found that participants with overweight swallowed more in response to the cues paired with chocolate milk, than to those paired with water. In lean participants, such cue discrimination was not revealed. Hence, mere confrontation with food or conditioned cues might lead to food cravings and be one precondition of overeating and binge eating behavior. Research on differences in being overweight and obesity regarding food cue reactivity mostly targets implicit cognitive processes (Svaldi et al., 2010; Loeber et al., 2012; Schag et al., 2013; Kollei et al., 2018). Altogether, these studies found exaggerated vigilance to food cues in individuals with overweight and obesity (for an overview, see Hendrikse et al., 2015). Furthermore, greater visual attention to high-energy density food images compared to pictures of low-energy density foods was found in participants with obesity (Doolan et al., 2014).

On a more behavioral basis, studies showed food cue exposure to increase cravings and prospective portion sizes for palatable foods (Ferriday and Brunstrom, 2008; Van den Akker et al., 2017) and revealed this effect to differ in participants with overweight or obesity compared to lean subjects (Tetley et al., 2009; Ferriday and Brunstrom, 2011; Ng and Davis, 2013). Meule et al. (2018) reported cue-induced food cravings to be significantly more prevalent in a group of patients with BED or bulimia nervosa compared to a healthy control group, with both groups showing normal weight. Research on cue-induced food craving in individuals with both, obesity and BED is sparse (see review by Hallam et al., 2016). For instance, Ng and Davis (2013) showed evidence for more cue-induced food cravings in individuals with obesity and BED. Compared to subjects with obesity without BED and lean controls, individuals with obesity with BED reported more pre- and post-craving following exposure to snack foods.

Since craving and cue reactivity were reported to be specific for highly palatable foods (Hill, 2007; Doolan et al., 2014), the theory of a conditioned incentive value of energy-dense food has been formulated (e.g., Havermans, 2013). Accordingly, positive associations of craving for energy-dense aliments with respective intake were shown for individuals with overweight and obesity (Chao et al., 2014; Myers et al., 2018). Supporting evidence was also found in studies investigating reward-related activation in response to palatable food cues (e.g., Lawrence et al., 2012; see review by de Macedo et al., 2016). For instance, Stoeckel et al. (2008) found greater activation in the nucleus accumbens, medial and lateral orbitofrontal cortex and other reward-related areas in individuals with obesity than in normal-weight individuals. Furthermore, increased activation of the nucleus accumbens, anterior cingulate, and insula was found to be predictive of less success in a weight loss program for individuals with obesity (Murdaugh et al., 2012). Altogether, these studies provide further insights into possible processes behind altered reactivity to highly palatable food cues in individuals with obesity, but little is known about how this specificity of cue reactivity and experienced craving accounts for individuals with obesity and BED.

Our main target was to assess cue-induced food craving in individuals with obesity with and without BED using a cue reactivity paradigm to explore differences in cravings at the sight of palatable food. We expected individuals with BED to show more cue-induced craving than individuals without BED. Moreover, we aimed to examine differences in cue-induced craving for categories of foods, expecting cues of High Fat High Sugar (HFHS) nourishments to elicit significantly more cue-induced food craving than those of Low Fat Low Sugar (LFLS) foods. In terms of the incentive value of sugar and sweet taste (Havermans, 2013), we hypothesized that cues of sweet HFHS foods in particular would induce the highest craving.

In addition to cue-induced cravings, the concepts of state and trait-like food cravings have also been investigated (Cepeda-Benito et al., 2000; Moreno et al., 2008). While state food craving describes the perceived intense desire for food in the moment of data collection, trait food craving describes the habitual aspect of food cravings. Trait food craving was shown to discriminate between patients with obesity with and without binge eating tendencies (White and Grilo, 2005; Innamorati et al., 2014). Altogether, food craving is a multidimensional construct with various aspects, differing in their stability over time (for reviews see Hallam et al., 2016; Meule, 2020). To the best of our knowledge, only a few studies have assessed trait and state food cravings using questionnaire data while at the same time examining differences in cue-induced food craving in individuals with obesity with and without BED (e.g., Ng and Davis, 2013). Therefore, this study additionally targeted differences in state and trait food cravings, hypothesizing food cravings to be greater in state and trait for individuals with obesity and BED than they are for individuals with obesity without BED.

## Materials and Methods

### Participants

In cooperation with Schönklinik Bad Bramstedt, 34 inpatients with obesity and binge eating disorder (BED) were recruited from the ward for patients with obesity and affective disorders. The inclusion criteria were a diagnosed binge eating disorder and a body mass index (BMI) of 30 and above. The exclusion criteria were severe mental or neurological illnesses (e.g., bipolar disorder, schizophrenia, dementia), a history of substance use disorder, current pregnancy, or a vegetarian/vegan diet. None of the participants had to be excluded due to these criteria. If subjects were using antidepressant medication, they were included in the study if the medication was stable for at least 2 weeks.

For the obese, non-binge eating disorder (N-BED) group, 38 participants were recruited via advertisements in social networks and posters in public places (asking for healthy volunteers with obesity) and screened via structured telephone interviews for the following exclusion criteria: BMI below 30, current pregnancy, vegetarian/vegan diet, BED, and severe mental illnesses as listed above. When invited to take part in the study, the German version of Structured Clinical Interview for DSM-IV screening and interview (SCID; Fydrich et al., 1997) were implemented by two trained psychologists and a senior psychologist (JR). In addition, the amount of binge eating episodes was assessed with the EDE-Q (Hilbert and Tuschen-Caffier, 2006) after the paradigm. Two participants of the N-BED group were excluded from the analysis because they reported a pathological amount of binge eating episodes. Hence, the final sample of the N-BED group consisted of 36 individuals with no one reporting more than 3 days of binge eating and/or inappropriate weight compensatory behavior (i.e., use of laxatives or vomiting for weight control).

The overall mean age of the participants was 36.27 years (SD = 11.50), and the overall mean BMI was 41.46 (SD = 8.05, range = 30.0–62.6). The two groups (BED vs. N-BED) differed in several characteristics (e.g., BMI, trait food craving, symptoms of eating disorders and depressive symptom severity), as shown in Table 1.

**Table 1:** Sample characteristics separated by group.

	**BED**		**N-BED**		
	***n*** **=** **34**		***n*** **=** **36**		**Test statistics**
	***(M=8, F=26)***		***(M=18, F=18)***		**$\chi_{\left(1\right)}^{2}$ = 5.25, *p* = 0.02, *d* = 0.569**
	**M**	**SD**	**M**	**SD**	
Age, y	41.41	12.00	31.42	8.64	*t*_(59.73)_ = −3.98, *p < *0.001, *d =* −0.96
BMI	46.16	8.28	37.03	4.64	*t*_(51.22)_ = −5.65, *p < *0.001, *d =* −1.37
BDI-II	26.26	14.03	6.94	6.06	*t*_(44.37)_ = −7.40, *p < *0.001, *d =* −1.81
EDE-Q total	3.54	1.05	2.06	0.96	*t*_(68)_ = −6.16, *p < *0.001, *d =* −1.47
Number of binge eating days in the past 28 days	8.03	8.433	1.03	1.70	*t*_(35.53)_ = −4.75, *p < *0.001, *d =* −1.17
FCQ-T total	163.18	34.29	108.22	33.10	*t*_(68)_ = −6.82, *p < *0.001^a^, *d =* −1.63
FCQ-S total *pre-paradigm*	38.12	13.85	27.03	10.86	*t*_(62.56)_ = −3.71, *p < *0.001^a^, *d =* −0.89
FCQ-S total *post-paradigm*	46.15	13.67	33.44	13.23	*t*_(68)_ = −3.95, *p < *0.001^a^, *d =* −0.95
Hunger	33.32	27.73	25.53	26.60	*t*_(68)_ = −1.20, *p =* 0.234

*M, male; F, female; BED, Subjects with Binge Eating Disorder and Obesity; N-BED, Subjects with Non-Binge Eating Disordered Obesity. M, mean; SD, standard deviation; BMI, Body Mass Index; BDI-II, Beck Depression Inventory II; EDE-Q, Eating Disorder Examination Questionnaire; FCQ-T, Food Cravings Questionnaire Trait; FCQ-S, Food Cravings Questionnaire State*.

a *p-values were Bonferroni-adjusted and are smaller than 0.001*.

The study adhered to the Declaration of Helsinki, and the ethics committee of the medical faculty of the University of Kiel and the medical council Schleswig-Holstein approved the study (D 459/18). All participants provided written informed consent.

### Questionnaires

The German version of the Food Cravings Questionnaire Trait (FCQ-T; Meule et al., 2012) measures the frequency and intensity of a person's food craving experiences in general. The questionnaire consists of 39 items that are scored on a six-point scale ranging from 1 = “never” to 6 = “always.” The FCQ-T comprises the Intentions, Positive Reinforcement, Negative Reinforcement, Lack of Control, Thoughts, Hunger, Emotions, Cues, and Guilt subscales. Higher scores indicate more food cravings.

The German version of the Food Cravings Questionnaire State (FCQ-S; Meule et al., 2012) measures the situationally perceived intense desire or food craving with 15 items that are assessed on a five-point scale ranging from 1 = “strongly disagree” to 5 = “strongly agree.” It comprises the subscales Desire, Positive Reinforcement, Negative Reinforcement, Lack of Control, and Hunger. Higher scores indicate a greater momentary perceived food craving.

The German version of the Eating Disorder Examination Questionnaire (EDE-Q; Hilbert and Tuschen-Caffier, 2006) measures pathological eating behaviors and related concerns. It comprises four subscales: Restraint, Eating Concern, Weight Concern, and Shape Concern. The EDE-Q has good convergent validity, and the questionnaire can differentiate between persons with and without eating disorders. Accordingly, a mean sum of 1.44 with *SD* = 1.22 is regarded as normal for subjects without eating disorders (Hilbert and Tuschen-Caffier, 2006), and higher scores indicate a more severe eating disorder pathology.

The German version of the Beck Depression Inventory Revised (BDI-II; Beck et al., 2001) is a questionnaire that measures depressive symptom severity. Four statements are given for each of the 21 symptom areas of depression. A score of 13 is regarded as critical for a mild severity of depression, 18 as medium and a score of 28 as severe depression.

### Design and Procedures

In both groups, participants were asked to arrive at the laboratory in a state of satiety to control for the effects of hunger on state food craving. Therefore, everyone agreed to eat a usual lunch *ad libitum* on the testing day and to refrain from eating until testing started 90 min later. Inpatients followed the diet of the hospital, which consisted of a suitable combination of macro- and micronutrients (e.g., meat with potatoes, vegetables and salad), and were also able to eat *ad libitum*. When arriving at our laboratory, the participants first reported their latest meal (e.g., curry with rice, soup with bread and meatballs) and then rated their perceived hunger on a visual analog scale (VAS; ranging from 0 = “not at all hungry” to 100 = “very hungry”). The results for these instructions are shown in Table 1. They then completed the computerized cue reactivity paradigm. To assess state food craving, we implemented the FCQ-S directly before (FCQ-S *pre*) and directly after (FCQ-S *post*) the cue reactivity paradigm. The FCQ-T as well as all other questionnaires (EDE-Q, BDI-II) were implemented after the paradigm. A researcher was present throughout the whole session to answer the participants' questions.

#### Food Cue Reactivity

**Figure 1:**
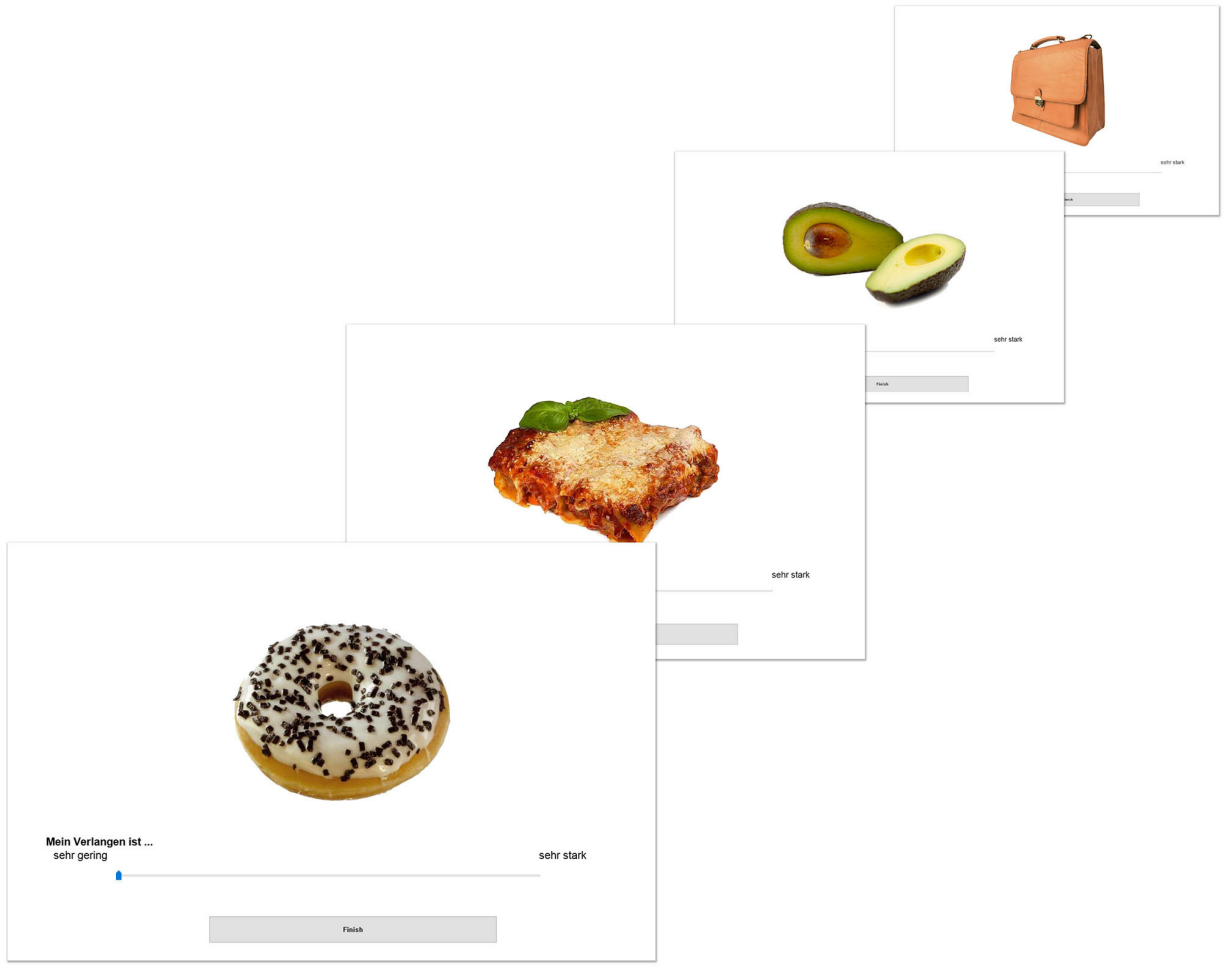
Examples of stimuli (HFHS sweet, HFHS savory, LFLS, and Non-Food) in our paradigm to assess cue-induced food craving. Pictures from the food-pics database (Blechert et al., 2014b).

The implemented paradigm is a modified version of the one described in Reents et al. (2020). We developed it to assess cue-induced food craving to pictorial stimuli. Participants view 200 pictures of three categories of food stimuli (High Fat and High Sugar, Low Fat and/or Low Sugar, and Non-Food) consecutively and rate their self-perceived craving. The category “High Fat and High Sugar” (HFHS) contains the two subcategories *sweet* and *savory* with 50 pictures each. Therefore, the 200 stimuli are dividable into four sets of 50 pictures each (Low Fat/Low Sugar, HFHS *sweet*, HFHS *savory*, and Non-Food). The HFHS *sweet* food pictures depicted sweet high-caloric food items (e.g., cake, chocolate). Likewise, pictures of the HFHS *savory* food category depicted savory high-caloric food items (e.g., pizza). Pictures in the Low Fat/Low Sugar (LFLS) food category included mostly unprocessed food items, which were palatable in the way presented (for instance no raw potatoes or uncooked rice). The 50 Non-Food stimuli depicted inedible objects (e.g., telephone, bicycle). We used pictures of the image database of experimental research on eating and appetite (Blechert et al., 2014b). Pictures were presented centered on a white background on a computer screen using Inquisit 4 (2015). Participants were asked to look at the picture and then rate their currently perceived craving to consume the shown food item. In the case of Non-Food stimuli, they were instructed to interpret the craving as the urge to engage with the item. The rating was made by moving the mouse pointer on a VAS, which indicated “my craving is” and ranged from “very low” to “very high.” Stimuli changed when the participant moved the indicator of the VAS and then hit the “finish” button underneath. Examples are shown in Figure 1.

### Statistical Analysis

All of our analyses were performed using IBM *SPSS* (2013). To test for group differences in demographics, questionnaire data and self-reported hunger, we conducted *t*-tests (see Table 1). To assess changes in state food craving that had occurred during testing, we conducted a mixed ANOVA with FCQ-S scores as the dependent variable, group as a between-subjects factor (BED vs. N-BED) and time (*pre-* and *post*-cue reactivity paradigm) as the repeated measures factor.

To realize cue-induced food craving as a dependent variable, we used the calculated means of the food craving ratings assessed in our paradigm. For our analysis of cue-induced food craving, we conducted a mixed ANOVA for our 2 × 3 design with group as a between-subjects factor with two conditions (BED vs. N-BED) and food category as a within-subjects factor with three conditions (Non-Food, LFLS, and HFHS).

For additional analyses, we divided the HFHS category into its subsets HFHS *sweet* and HFHS *savory* and conducted a 2 × 2 ANOVA with group as a between-subjects factor (BED vs. N-BED) and subsets as a within-subjects factor with two conditions (HFHS *sweet* and HFHS *savory*).

Since we found significant group differences regarding age, BMI, and depression symptom severity (see Table 1), mixed ANCOVAs were conducted with group as the between-subjects factor (BED vs. N-BED), food category as the within-subjects factor (HFHS, LFLS) adjusted for age, BMI and BDI-II scores as the covariates, respectively.

To estimate the effects of depression symptom severity on food cravings, we calculated the Pearson correlation for the relation of BDI-II scores with cue-induced craving scores from our paradigm, FCQ-S and FCQ-T scores. Additionally, we calculated Pearson correlations for cue-induced craving scores with FCQ-S and FCQ-T sum scores.

To estimate the relationships of cue-induced cravings with trait and state food cravings, we calculated Pearson correlations of cue-induced craving scores for the categories HFHS, LFLS, and Non-Food with scores in FCQT, FCQS *pre*-paradigm and FCQ-S *post-*paradigm. The correlation coefficients were compared using Fisher's *z-*tests implemented in the R toolbox cocor (Diedenhofen and Musch, 2015).

When required, degrees of freedom were adjusted with the Greenhouse-Geisser correction for non-sphericity. For significant effects in our analysis, planned *post-hoc t*-tests were conducted. Significance was assumed at *p* < 0.05.

## Results

### Cue-Induced Food Craving

**Figure 2:**
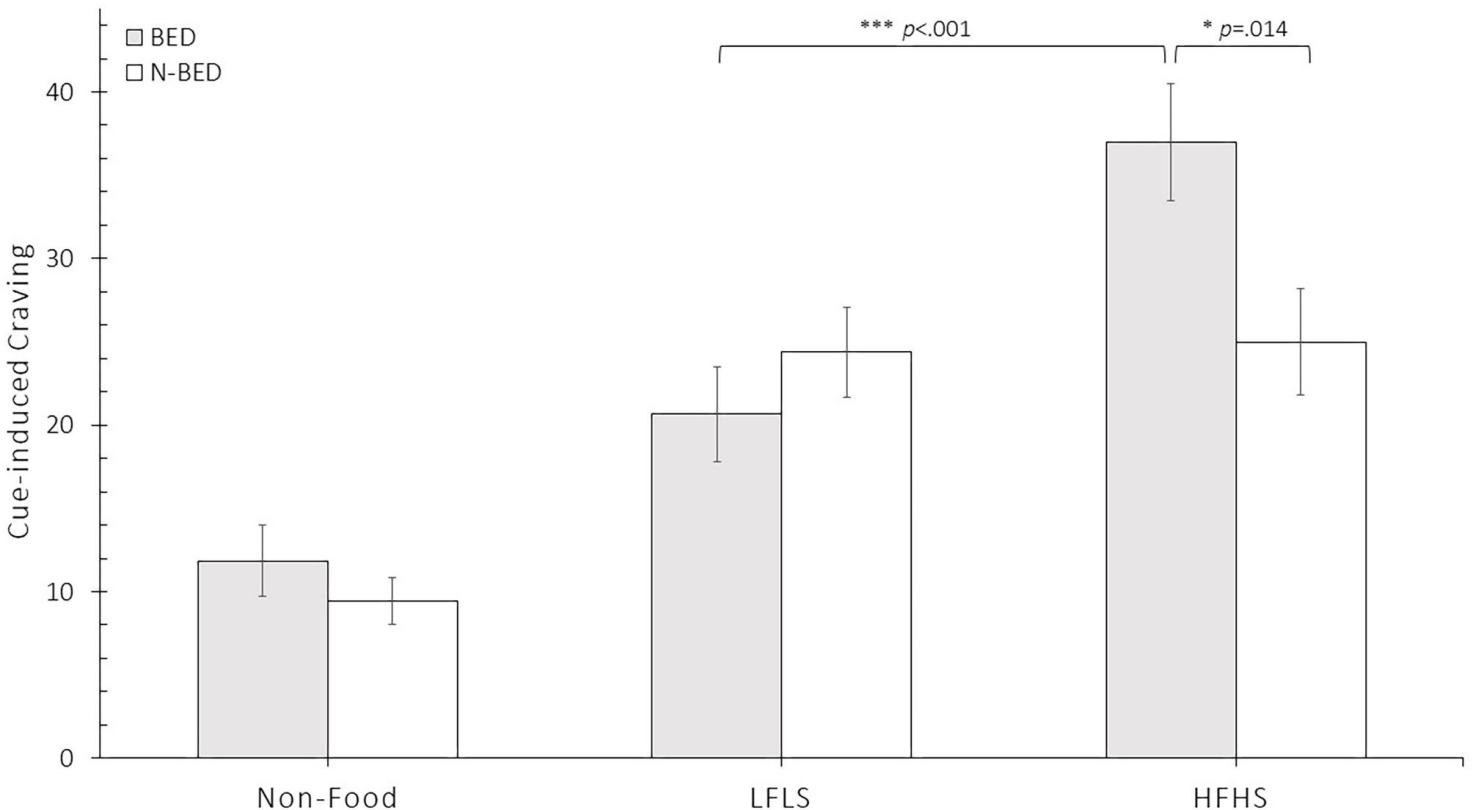
Results of mean food craving (± SEM) separated for the group of individuals with obesity and binge eating disorder (BED) and the group of individuals with obesity without eating disorders (N-BED) in the three main categories of stimuli: Non-Food, low fat/low sugar (LFLS) and high fat and high sugar (HFHS). *p < 0.05, ***p < 0.001.

We conducted a mixed ANOVA with group as a between-subjects factor (BED vs. N-BED) and food category as a within-subjects factor (Non-Food, LFLS, and HFHS). The analysis revealed a significant interaction of group and category, *F*_(1.80, 122.52)_ = 6.72, *p* = 0.002, η^2^ = 0.090, indicating that the two groups revealed differences in craving with regard to the three categories Non-Food, LFLS, and HFHS. Planned *post-hoc t*-tests revealed that participants in the BED group compared to the N-BED group showed significantly more cue-induced cravings for the HFHS category, *t*_(68)_ = −2.53, *p* = 0.014, *d* = −0.605, but not for the LFLS category, *t*_(68)_ = 0.953 *p* = 0.344. As expected, no significant difference between BED and N-BED was found for the Non-Food category, *t*_(56.98)_ = −0.95 *p* = 0.347. There was a significant main effect of category, *F*_(1.80, 122.52)_ = 44.78, *p* < 0.001, η^2^ = 0.397, but no significant main effect of group, *F*_(1, 68)_ = 1.50, *p* = 0.225. Further analysis revealed significantly more cue-induced cravings for the category HFHS compared to LFLS, *t*_(33)_ = 4.73, *p* < 0.001, *d* = 0.873 in BED, but not in N-BED, *t*_(35)_ = 0.248, *p* = 0.805 (see Figure 2). An ANCOVA on food category (HFHS and LFLS) and group (BED vs. N-BED) with age as a covariate revealed no significant main effect or interaction of age. The interaction of group and food category remained significant, *F*_(1, 67)_ = 14.09, *p* < 0.001, η^2^ = 0.174. Adding BDI-II as covariate showed no significant main effect or interaction effects for BDI-II, but the interaction of food category and group remained significant *F*_(1, 66)_ = 4.45, *p* = 0.039, η^2^ = 0.063. Adjusting for age and BMI revealed no significant main effects or interaction effects for age and BMI, but again, the interaction of food category and group stayed significant *F*_(1, 66)_ = 8.89, *p* = 0.004, η^2^ = 0.119.

To further analyze the group differences in the HFHS category, we divided this category into its subsets *sweet* and *savory* (type of HFHS). A 2 × 2 ANOVA revealed a significant main effect of group, *F*_(1, 68)_ = 6.418, *p* = 0.014, η^2^ = 0.086, but no significant main effect of type of HFHS, *F*_(1, 68)_ = 0.042, *p* = 0.839 or interaction, *F*_(1, 68)_ = 1.272, *p* = 0.263.

### Trait Food Craving

We conducted a *t*-test to compare FCQ-T scores of the BED and N-BED groups, which showed significantly higher trait food craving scores in the BED group (see Table 1).

### State Food Craving

We conducted a mixed ANOVA with the between-subjects factor group (BED vs. N-BED) and the within-factor time (pre- and post-paradigm) on state food craving (FCQ-S scores). The analysis revealed a significant main effect of group with *F*_(1, 68)_ = 18.41, *p* < 0.001, η^2^ = 0.213 and a significant main effect of time with *F*_(1, 68)_ = 27.09, *p* < 0.001, η^2^ = 0.289 but no significant interaction, *F*_(1, 68)_ = 0.34, *p* = 0.56. Planned *post-hoc t*-tests revealed significantly higher FCQ-S scores in the BED group than in the N-BED group (BED: *t*_(33)_ = −4.43 *p* < 0.001, *d* = −0.583; N-BED: *t*_(35)_ = −3.13, *p* = 0.003, *d* = −0.525), indicating that food stimuli presented on the computer screen increased state craving in both groups (see Table 1).

### Correlational Analyses

#### Associations Between the Three Measures of Food Craving

In the BED group, behaviorally assessed cue-induced craving for HFHS was positively associated with trait as well as state food craving, whereas LFLS was only associated with trait craving. Additionally, in the N-BED group cue-induced craving for HFHS was positively related to trait and state craving. The magnitude of these correlations did not differ significantly between BED and N-BED group (Fisher's *z*-tests all *p*'s > 0.223). The results are shown in Table 2.

**Table 2:** Correlations of cue-induced cravings in the BED and N-BED groups.

	**BED**			**N-BED**		
	**HFHS**	**LFLS**	**Non-Food**	**HFHS**	**LFLS**	**Non-Food**
FCQ–T	0.666^**^	0.345^*^	−0.195	0.583^**^	0.384^*^	0.347^*^
FCQ-S *pre*	0.457^**^	0.304	−0.216	0.371^*^	0.420^*^	0.285
FCQ-S *post*	0.665^**^	0.270	−0.238	0.545^**^	0.294	0.091

*HFHS, cue-induced craving scores for high-fat and high-sugar food; LFLS, cue-induced craving scores for low-fat/low-sugar food; FCQ-T, Sum Score Food Cravings Questionnaire Trait version; FCQ-S, Sum Score Food Cravings Questionnaire State version*.

* *p < 0.05*,

** *p < 0.01*.

#### Associations Between Food Craving Measures and Depression

In the BED group that partly exhibited relevant depressive symptoms, we did not find any significant correlations between the severity of depression and cue-induced craving, ρ_*BDI, HFHS*_ = 0.278, *p* = 0.112; ρ_*BDI, LFLS*_ = 0.098, *p* = 0.583; ρ_*BDI, Non*−*Food*_ = −1.89, *p* = 0.286. However, there were moderate positive correlations between depression and state as well as trait food craving (ρ_*BDI*_,_*FCQ*−*S*_ = 0.43, *p* = 0.012; ρ_*BDI, FCQ*−*T*_ = 0.48, *p* = 0.004). In the N-BED group that showed a significantly lower degree of depressive symptoms, no significant correlations with craving measures were revealed (all *p*'s > 0.14).

## Discussion

In the current study, we explored differences in food craving in individuals with obesity with and without BED using a food cue reactivity paradigm and questionnaire data. We found food cravings to be more prevalent in individuals with BED in all three assessments—cue-induced, state, and trait food cravings—and moreover, these different aspects of food craving were interrelated at a moderate level.

### Cue-Induced Food Craving

In participants with obesity and BED, cue-induced craving was specifically increased for HFHS foods compared to individuals without BED. This is in line with findings of altered food cue reactivity for calorie-dense foods in individuals with obesity (Stoeckel et al., 2008; Stice et al., 2009; Mehta et al., 2012; Zhang et al., 2018), resulting in greater self-perceived cravings for those aliments (Jansen, 1998; Havermans, 2013; Ng and Davis, 2013). However, contrasting sweet and savory HFHS food did not reveal further differential effects between persons with obesity with and without BED. In light of incentive values (cf. Havermans, 2013) anticipated, sweet taste seems to be less relevant than the level of carbohydrates and fat. This supports findings of fat being the relevant macronutrient resulting in heightened body weight (for meta-analysis see Hooper et al., 2020), whereas sucrose consumption seems to be less relevant for obesity (e.g., Parnell et al., 2008). Moreover, it might also give further implications about the hypothesis that a combination of fat and carbohydrates is more relevant for an incentive value than the anticipation of a sweet taste (DiFeliceantonio et al., 2018).

Etiologically, our results for cue-induced craving are in line with the idea of heightened food- cue reactivity leading to heightened self-perceived craving (Jansen, 1998; Ferriday and Brunstrom, 2008; Boswell and Kober, 2016), which in turn might facilitate binge eating behavior (Ng and Davis, 2013; Meule et al., 2018). Therefore, our results on heightened cue-induced craving in individuals with BED support the conditional model of binge eating proposed by Jansen (1998). Hence, this study provides further evidence that craving and excessive HFHS food intake in individuals with obesity and BED might be cue controlled.

### Trait Food Craving

In line with findings of heightened trait food craving in individuals with obesity and binge eating symptomatology (White and Grilo, 2005; Innamorati et al., 2014; Meule et al., 2014a, Meule et al., 2017), participants with obesity and BED reported more trait food cravings than those without BED. Of note, we did not explicitly recruit a selective control sample with high trait food craving scores. We included a naturalistic control group, following the idea that trait food craving might be crucial when distinguishing individuals with obesity and BED from individuals with obesity but without BED (e.g., Innamorati et al., 2014).

### State Food Craving

As reported in previous studies (e.g., Meule et al., 2018) state food craving assessed with the FCQ-S was higher in individuals with binge eating symptomatology than in the control group. In line with findings by Ng and Davis (2013), state food craving in our sample was higher in participants with BED not only after cue-exposure but also at a baseline measure. Hence, state food cravings seem to be sensitive to situational changes such as external food cues, even if these are only pictorial (Brignell et al., 2009; Blechert et al., 2014a; Meule et al., 2014b).

### Depression Symptom Severity and Food Cravings in BED

In the present study, we only found associations of the depression symptom severity with state and trait food craving, but not with cue-induced craving in individuals with BED. As negative cognitions, i.e., body dissatisfaction, dietary mentality, restrictions and distress are strongly associated with binge eating behavior (for a review see Nicholls et al., 2016), it is not clear whether dysphoric or depressive mood promotes binge eating (Spoor et al., 2006) or whether binge eating might contribute to the development of depressive mood (Johnson and Wardle, 2005; Haedt-Matt and Keel, 2011). In the light of these findings, altered mood might generate a state in which individuals become more vulnerable and responsive which, in turn, might result in heightened food craving. Accordingly, negative mood states have been reported to increase eating behavior, an effect described as emotional eating (e.g., Macht, 2008; Bongers et al., 2015). For instance, emotional eating was revealed in individuals with binge eating symptomatology (Telch and Agras, 1996; Schulz and Laessle, 2010) and binge eating behavior has been suggested to be a dysfunctional emotion regulation strategy (Nicholls et al., 2016; for a review see Dingemans et al., 2017). Even in participants with obesity without eating disorder, a high negative affect type was associated with cue-induced overeating (Jansen et al., 2008). However, in our study, cue-induced food cravings were higher in participants with BED than in the N-BED group even when adjusting for the level of depressive symptoms. Nevertheless, the relationship between negative states of mood, binge eating, and food cravings should be addressed in future research.

There are some limitations to our study. First, our study examined self-reported food craving, which is thought to be elicited by physiological responses to food cues (Wardle, 1990; Jansen, 1998; Nederkoorn et al., 2004). To further understand the physiological basis of cravings for specific nourishments, i.e., HFHS and LFLS, it might be beneficial to combine our behavioral assessment of craving with a measurement of physiological parameters (e.g., García-García et al., 2013; Blechert et al., 2014a; Hume et al., 2015). Second, as LFLS stimuli depicted low processed food and HFHS depicted highly processed food, differences in craving between these two food categories might reflect not only caloric content but also the level of processing. Third, participants with BED were older than those without BED. However, age differences are unlikely to account for our results, as covariation analysis revealed no effect of age, and age seems to have a decreasing effect on food craving (Pelchat and Schaefer, 2000) as well as food cue reactivity (Charbonnier et al., 2018). Finally, in the BED group the proportion of women was higher than in the N-BED group. Hence, gender distribution might have influenced our results. In a recent review, Hallam et al. (2016) reported men to crave more for savory food, whereas women crave more for sweet food. However, in our sample, no group-related differences in cue-induced craving regarding sweet vs. savory food were revealed.

In the present study, trait, state and cue-induced food cravings were significantly more prevalent in individuals with obesity with BED than in individuals with obesity without BED. The fact that heightened cue-induced craving was revealed for all food stimuli over Non-Food stimuli reflects that food craving can be convincingly induced and measured by implementing pictorial stimuli (cf., Ledoux et al., 2013) in individuals with obesity with and without BED. Specifically, the intensity of cue-induced cravings for HFHS aliments was higher in individuals with obesity and BED. Given our obesogenic environment in which food stimuli appear in daily life through advertisements, television and electronic media, cue-induced craving might be of great relevance for understanding the role of overeating and binge eating in the development of overweight and obesity. This is supported by findings that watching television (Kaur et al., 2003; Harris et al., 2009; Zimmerman and Bell, 2010) and fast food restaurant availability (Ledoux et al., 2015) are associated with higher weight status and BMI.

Our findings are in line with the conditioned binge eating model proposed by Jansen (1998) and therefore support the idea, that high relapse rates in the treatment of BED might reflect virtually unchanged cue reactivity levels for HFHS food. Hence, it might be beneficial to include aspects of cue-induced craving in the therapeutic approaches such as cue exposure and response prevention in patients with obesity and BED (cf., Jansen et al., 2016; Schyns et al., 2018; Akker et al., 2020).

## Data Availability Statement

The raw data supporting the conclusions of this article will be made available by the authors, without undue reservation.

## Ethics Statement

The studies involving human participants were reviewed and approved by ethics committee of the medical faculty of the Christian-Albrechts-University of Kiel and the Medical Counsil Schleswig-Holstein (D 459/18). The patients/participants provided their written informed consent to participate in this study.

## Author Contributions

JR and AP designed the study and prepared the final version of the manuscript. JR conducted literature searches, provided summaries of previous research studies, acquired the data, and performed the statistical analyses. AP was supervisor of this project. All authors contributed to the first draft of the manuscript and article and approved the submitted version.

## Conflict of Interest

The authors declare that the research was conducted in the absence of any commercial or financial relationships that could be construed as a potential conflict of interest.
